# Targeting regulatory T cells in glioblastoma: from mechanistic insights to novel immunotherapeutic strategies

**DOI:** 10.3389/fimmu.2026.1759023

**Published:** 2026-04-10

**Authors:** Jingwen Li, Yang Liu, Peng Peng, Jinyang Hu

**Affiliations:** 1 Department of Oncology, Xiangyang Central Hospital, Affiliated Hospital of Hubei University of Arts and Science, Xiangyang, China; 2 Department of Neurosurgery, Xiangyang Central Hospital, Affiliated Hospital of Hubei University of Arts and Science, Xiangyang, China; 3 Wenzhou Medical University, Wenzhou, China; 4 Department of Neurosurgery, The Second Affiliated Hospital of Wenzhou Medical University, Wenzhou, China

**Keywords:** clinical translation, glioblastoma, immune evasion, immune microenvironment, immunotherapy, regulatory T cells, targeted therapy

## Abstract

Glioblastoma (GBM), the most common and aggressive primary brain tumor in adults, remains a formidable therapeutic challenge. Within the immunosuppressive tumor microenvironment, regulatory T cells (Tregs) have attracted increasing attention for their pivotal role in facilitating tumor immune evasion and sustaining immunosuppression. Through diverse mechanisms, Tregs potently inhibit anti-tumor immunity, thereby driving tumor progression and contributing to therapeutic resistance, which collectively correlates with poor clinical outcomes. This review systematically outlines the biological features and regulatory networks of Tregs in GBM, with particular emphasis on emerging strategies designed to target these cells. We discuss approaches such as Treg depletion, interference with their recruitment, functional reprogramming, and combination immunotherapies. Furthermore, we critically assess the translational progress and clinical limitations of these approaches, including challenges related to target specificity, immune adaptation, and treatment-related toxicities. By synthesizing mechanistic insights with therapeutic prospects, this review aims to inform future directions in precision immunotherapy and inspire multidisciplinary efforts toward effective Treg-targeting regimens for GBM.

## 1 Introduction

Glioblastoma (GBM), the most common and aggressive primary brain tumor in adults, portends a dismal prognosis with a median survival of merely 12 to 15 months. Despite aggressive standard care-including maximal safe resection, radiotherapy, and temozolomide chemotherapy-long-term survival remains elusive (1, 2). This therapeutic impasse stems not only from GBM’s invasive and heterogeneous nature but also from the profoundly immunosuppressive tumor microenvironment (TME) that subverts both innate and adaptive anti-tumor immunity.

Within this complex TME, regulatory T cells (Tregs) have emerged as central orchestrators of local immune suppression. GBM recruits and sustains a high density of Tregs (3, 4), which potently inhibit effector T-cell function through multiple contact-dependent and cytokine-mediated mechanisms, such as secretion of TGF-β and IL-10, expression of immune checkpoints including PD-L1, and metabolic disruption of local interleukin-2 availability (5–7). These activities are further reinforced through crosstalk with tumor-associated macrophages and myeloid-derived suppressor cells, establishing a resilient immunosuppressive network that facilitates tumor immune escape and underlies resistance to conventional and immunotherapeutic interventions (8, 9).

Growing recognition of Tregs’ pivotal role has stimulated the development of strategies aimed at their selective targeting. Current investigative approaches include Treg-depleting antibodies against surface markers like CTLA-4 (10), inhibition of Treg trafficking, functional interference using small molecules or gene editing, and combination regimens with checkpoint inhibitors or cancer vaccines (11–13). However, a critical challenge persists in achieving sufficient Treg suppression within the tumor without inducing systemic autoimmunity (14, 15). Deeper insight into the molecular mechanisms governing Treg recruitment, stability, and function in GBM is therefore essential to guide the rational design of next-generation immunotherapies.

This review systematically examines the biology of Tregs in GBM, their interplay with other components of the TME, and the evolving therapeutic strategies aimed at overcoming their immunosuppressive functions. By integrating mechanistic understanding with clinical translation, we aim to outline a path toward more precise and effective Treg-targeting regimens for this devastating disease.

## 2 Biology of Tregs in the glioblastoma microenvironment

### 2.1 Phenotypic characteristics and heterogeneity of Treg cells

Tregs are a specialized CD4+ T cell subset tasked with maintaining immune homeostasis and preventing autoimmunity. Their identity and suppressive function are classically defined by the expression of the transcription factor FoxP3 and the high-affinity interleukin-2 receptor alpha chain (CD25). FoxP3 acts as the master regulator, directing Treg development and functional programming, and remains the definitive marker for this lineage (16, 17). However, Tregs are not a uniform population. Advances in single-cell technologies, such as high-dimensional flow cytometry and transcriptomic sequencing, have unveiled a striking degree of phenotypic and functional heterogeneity within Tregs, reflecting adaptations to specific tissue contexts and immunological challenges (18, 19).

This heterogeneity is particularly evident in the GBM microenvironment. A subset of Tregs expressing the integrin CD103 (αEβ7) is notably enriched in GBM tissues. As a marker of tissue-resident memory T cells, CD103 facilitates the firm retention of Tregs within the tumor parenchyma, and its expression is associated with potent immunosuppressive activity (20, 21). Beyond the core Treg signature (FoxP3+ CD25+), CD103+ Tregs often display an upregulated profile of co-inhibitory receptors, including CTLA-4 and PD-1, underscoring their enhanced capacity for immune regulation (22, 23). Their adaptation to the tumor niche is further supported by distinct metabolic rewiring, such as shifts in lipid metabolism, which promotes their survival and functional fitness within the nutrient-poor and hypoxic GBM milieu (21).

The diversity of Tregs in GBM extends beyond the CD103+ subset and encompasses their developmental origin and spatial distribution. Two primary subsets—thymus-derived natural Tregs (nTregs) and peripherally induced Tregs (iTregs)—coexist in tumors and can be partially distinguished by markers like Neuropilin-1 (Nrp1) and Helios (24). These subsets may play non-redundant roles; nTregs are often implicated in establishing early immune tolerance, while iTregs might expand later in response to chronic antigen exposure. Spatially, Treg infiltration is not uniform. The invasive margin of GBM often exhibits a particularly high Treg-to-CD8+ T cell ratio, creating a potent barrier against cytotoxic T cell entry and function, whereas the tumor core may harbor Tregs with distinct, tissue-adapted phenotypes (25).

Moreover, the colloquial terms “positive” and “negative” Tregs, while not formal immunological classifications, are often used to describe two critical dimensions of Treg biology: marker expression and functional state. In the context of surface marker expression, “positive” typically denotes the presence of defining molecules such as CD25, FoxP3, or CTLA-4, which are associated with their regulatory identity and suppressive capacity. Conversely, the absence of certain markers can also define a “negative” phenotype that correlates with suppressive function. More importantly, these terms frequently allude to the profound functional heterogeneity and plasticity within the Treg compartment. In this sense, “positive” Tregs refer to stable, canonical suppressor cells that maintain immune tolerance. In GBM, a prime example is the CD103+ Treg subset, which is highly enriched in tumors, exhibits potent immunosuppressive activity, and is associated with resistance to therapy (26). In contrast, “negative” Tregs may describe unstable or contextually reprogrammed cells that can lose their suppressive function and acquire pro-inflammatory properties. Under inflammatory conditions, some Tregs can downregulate FoxP3 and produce effector cytokines like IFN-γ or IL-17, transitioning into Th1-like or Th17-like phenotypes that may exacerbate inflammation and contribute to disease pathology (27). This functional conversion blurs the line between regulation and inflammation, representing a “negative” outcome in terms of disease progression.

Adding another layer of complexity, this inherent plasticity, coupled with the marked heterogeneity of Treg subsets, complicates the precise identification of tumor-promoting Tregs based on a limited set of surface markers. Therefore, therapeutic strategies must move beyond a binary view. The goal is to precisely target the “positive” tumor-promoting Treg subsets (28) while sparing or even reinforcing the “positive” Tregs essential for systemic homeostasis, and to prevent the generation of pathogenic “negative” pro-inflammatory Treg derivatives. Achieving this will require integrating transcriptional, metabolic, and spatial data for the rational design of therapies that can selectively dismantle immunosuppressive networks in GBM without breaching systemic immune tolerance.

### 2.2 Mechanisms of Treg recruitment and infiltration in GBM

The recruitment and infiltration of Tregs into the GBM microenvironment are driven by three interconnected mechanisms: chemokine-directed migration, metabolic regulation, and cell-cell signaling.

Chemokine gradients play a fundamental role in directing Treg trafficking to the tumor site. Specific axes, such as CCL22-CCR4 and CCL1-CCR8, are critically involved (29, 30). GBM cells and tumor-associated stromal components secrete a range of chemokines, including CCL2 and CCL22, establishing a concentration gradient that guides Tregs into the tumor parenchyma (31). Evidence from canine high-grade glioma models confirms that disrupting the CCL2-CCR4 axis effectively curtails Treg migration and mitigates local immunosuppression (32). This recruitment is further amplified by a positive feedback loop, wherein initial Treg contact with tumor cells can upregulate chemokine production, thereby enhancing subsequent Treg influx.

While chemokine gradients are fundamental for directing Treg trafficking to the GBM site, their recruitment and functional integration into the tumor niche are critically coordinated by a complementary network of cytokines. Pro-inflammatory cytokines such as TNF-α can induce tumor and stromal cells to secrete higher levels of chemokines like CCL22, thereby amplifying the initial chemotactic signal for Treg recruitment (28, 33). More importantly, once Tregs are recruited, their stability, survival, and suppressive function are heavily dependent on specific cytokines within the TME. TGF-β is a master regulator that promotes the induction, FoxP3 expression, and functional stability of Tregs, preventing their conversion into inflammatory phenotypes (34). IL-10, often produced by Tregs themselves or by tolerogenic plasmacytoid dendritic cells (pDCs), serves as both a mediator and an enhancer of Treg-mediated suppression (35). Additionally, Tregs constitutively express the high-affinity IL-2 receptor (CD25), enabling them to competitively consume IL-2 in the microenvironment. This “cytokine deprivation” not only supports Treg survival but also critically restricts the expansion and function of effector T cells, constituting a key contact-independent immunosuppressive mechanism (36, 37). Thus, cytokines operate in concert with chemokines: chemokines guide Tregs to the tumor, while cytokines then shape their fate, maintenance, and functional output, establishing a resilient and self-reinforcing immunosuppressive circuit in GBM.

Beyond chemotaxis, the unique metabolic landscape of GBM actively facilitates Treg recruitment and functional adaptation. As a highly glycolytic tumor, GBM accumulates high concentrations of lactate in its microenvironment. Far from being a waste product, lactate functions as a signaling molecule that promotes immunosuppression. It induces histone H3K18 lactylation, which upregulates the expression of key immunosuppressive genes in Tregs, including CD39, CD73, and CCR8 (38). Interventions that disrupt lactate production or signaling have been shown to weaken Treg-mediated suppression and synergize with immunotherapy (39). This metabolic reprogramming allows Tregs to not to survive but to thrive in the otherwise hostile, hypoxic, and nutrient-poor tumor niche (40).

### 2.3 Mechanisms underlying Treg activation and functional maintenance

Regulatory T cells (Tregs) play a central role in maintaining immune tolerance and suppressing excessive immune responses. Their activation and functional sustainability are regulated through multiple interconnected mechanisms, including immune checkpoint signaling, epigenetic modifications, and metabolic reprogramming. A deeper understanding of these regulatory networks is essential for deciphering immune evasion in GBM and developing effective Treg-targeted immunotherapies.

Immune checkpoint molecules such as CTLA-4, PD-1, and GITR critically influence Treg activity. CTLA-4, highly expressed on Tregs, binds to CD80/CD86 on antigen-presenting cells, thereby limiting co-stimulatory signals and enhancing immunosuppression (41). PD-1 expression further strengthens Treg-mediated suppression (42), and PD-1hi Tregs are enriched in the glioma microenvironment, facilitating tumor immune escape (43). GITR activation exerts dual effects on Tregs, modulating both their expansion and suppressive function (44). Recent studies also reveal cross-regulation among checkpoint pathways: DNAM-1 competes with TIGIT for CD155 binding, and loss of DNAM-1 enhances TIGIT signaling, which suppresses mTORC1 activity and helps maintain Foxp3 expression and Treg stability (45).

Epigenetic mechanisms are fundamental in preserving Treg lineage identity and functional integrity. DNA methylation, governed in part by the maintenance methyltransferase Uhrf1, tightly controls Foxp3 expression and other immunosuppressive genes. Uhrf1 deficiency leads to DNA hypomethylation, triggering inflammatory gene programs and compromising Treg stability (46). Additional chromatin modifications, including histone deacetylation, further fine-tune Treg adaptability within the tumor microenvironment (47). Key transcription factors also contribute: BACH2 promotes the quiescence and long-term maintenance of resting Tregs by competing with AP-1 for binding sites (48), while JunB regulates effector programs in tissue-resident Tregs (49). The nuclear receptor Esrrg helps maintain mitochondrial homeostasis, and its loss disrupts Treg metabolism and function, leading to autoimmunity (50).

Metabolic pathways likewise underpin Treg activation and functional persistence. MALT1 protease activity bridges TCR signaling to MYC-driven mitochondrial biogenesis and proliferation; deficiency in either MALT1 or Myc impairs Treg fitness and causes autoimmunity (51). The AMPK–SIRT2 axis acts as an energy-sensing pathway that supports Treg function, and loss of VPS34 perturbs metabolic homeostasis and suppressive capacity (52). mTOR signaling serves as a metabolic checkpoint: its inhibition helps sustain Foxp3 expression and Treg function, whereas its activation can weaken immunosuppressive activity (44, 45). Moreover, lactate derived from the tumor microenvironment enhances Treg suppression via histone lactylation, which upregulates ectonucleotidases CD39 and CD73 and promotes immune evasion (53, 54).

In summary, Treg activation and functional maintenance depend on a multilayered regulatory network encompassing immune checkpoints, epigenetic programming, and metabolic adaptation. These mechanisms are deeply intertwined within the GBM immune microenvironment, where they collectively reinforce Treg-mediated immunosuppression and facilitate tumor immune escape. Unraveling these complex interactions will provide a mechanistic foundation for novel Treg-directed therapies, offering a promising avenue to disrupt the immunosuppressive barrier in GBM and improve treatment outcomes.

### 2.4 Interactions between Tregs and other immune cells in the tumor microenvironment

Tregs within the TME interact with other immune cells through multiple mechanisms, forming an immunosuppressive network that promotes tumor immune escape and progression. Tregs significantly suppress the function of CD8^+^ effector T cells and natural killer (NK) cells, thereby impairing antitumor immunity. Studies indicate that Tregs competitively consume critical cytokines such as interleukin-2 (IL-2), leading to functional exhaustion of CD8^+^ T cells (36). For instance, in prostate cancer, macrophages facilitate the co-aggregation of CD8^+^ T cells and Tregs via the CXCL12/CXCR4 axis; Tregs further exacerbate CD8^+^ T cell dysfunction by depleting IL-2 and mediating the IL-2/STAT5 signaling pathway, fostering an immunosuppressive microenvironment (37). In lung cancer, accumulation of CCR8^+^ Tregs suppresses the cytotoxicity of GzmB^+^ CD8^+^ T cells, correlating with poor patient prognosis (55). Additionally, lactate derived from tumor metabolism enhances Treg stability and function through MOESIN lactylation, indirectly inhibiting the antitumor activity of NK cells (56).

Tregs further reinforce immunosuppression by modulating the functions of tumor-associated macrophages (TAMs) and dendritic cells (DCs). In the TME, close interactions exist between Tregs and TAMs: TAMs not only recruit Tregs via secretion of chemokines such as CCL22 but also polarize toward an M2 phenotype, supporting tumor growth and immune evasion (57). In clear cell renal cell carcinoma, tumor-derived TGFBI upregulates CCL expression to promote Treg migration, enhancing immunosuppression and driving tumor progression (58). Tregs and DCs engage in bidirectional regulation: Tregs inhibit DC maturation and antigen-presenting capacity, while DCs support Treg activation and expansion through antigen presentation-a mechanism reported across multiple tumor microenvironments (59, 60). For example, in hepatocellular carcinoma, Tregs and cDC2 collaboratively suppress immune responses under hypoxic conditions, reducing antigen presentation efficiency and promoting immune escape (61).

Moreover, Tregs contribute to a complex immune escape network through immune checkpoint molecules. Tregs highly express immune checkpoint proteins such as CTLA-4, PD-1, and TIGIT, which not only regulate their own expansion and function but also inhibit effector T cell activity (62). Within TME, Tregs employ CTLA-4 mediated negative feedback to modulate the expression of co-stimulatory molecule B7, maintaining numerical stability. Although CTLA-4 blockade may expand Treg populations, effective antitumor responses require concomitant inhibition of Treg function (63). Furthermore, studies show that the long non-coding RNA HULC promotes immune escape by regulating the Treg/PD-1 axis, suggesting therapeutic potential in targeting Treg-associated immune checkpoint pathways (64).

**Figure 1:**
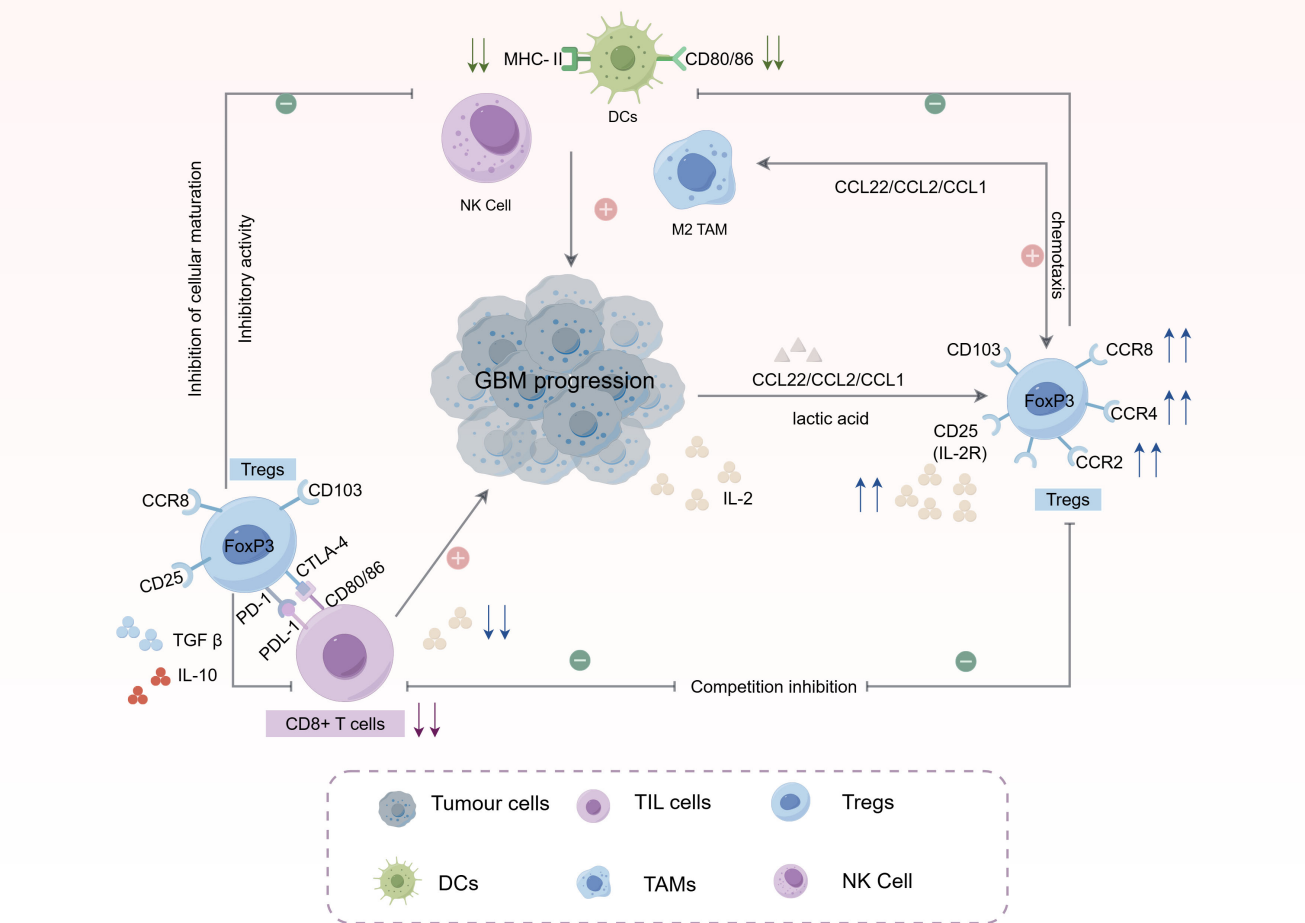
Multifaceted immunosuppressive mechanisms of regulatory T cells in the glioblastoma immune microenvironment. Tregs employ a variety of direct and indirect mechanisms to suppress antitumor immunity. These include: Direct suppression of effector T cells via inhibitory receptors (CTLA-4, PD-1) and cytokine secretion (TGF-β, IL-10); Metabolic disruption by consuming interleukin-2 (IL-2), leading to cytokine deprivation of CD8^+^ T cells, and responding to tumor-derived lactate which enhances their suppressive capacity via histone lactylation; Interaction with and modulation of other immune cells, such as inhibiting dendritic cell (DC) maturation, polarizing tumor-associated macrophages (TAMs) towards an M2 phenotype, and suppressing natural killer (NK) cell cytotoxicity. This collaborative network establishes a highly immunosuppressive niche that promotes tumor progression and therapy resistance.

These mechanisms not only elucidate the pivotal role of Tregs in tumor immunoregulation but also provide a theoretical foundation and clinical translation for immunotherapeutic strategies targeting Tregs and their associated pathways (**Figure 1**).

## 3 Strategies for targeting regulatory T cells

### 3.1 Treg depletion strategies: targeting surface markers and signaling pathways

Depleting Tregs has become a significant research focus in tumor immunotherapy. The core objective is to achieve selective elimination or functional reprogramming of Tregs by targeting surface markers or key signaling pathways, thereby enhancing antitumor immune responses. One promising approach involves the use of anti-CD25 antibodies to deplete intratumoral Tregs. CD25 (the IL-2 receptor α chain) is highly expressed on tumor-infiltrating Tregs. A non-IL-2-blocking anti-CD25 antibody (anti-CD25NIB) specifically reduces intratumoral Treg numbers while promoting clonal expansion of CD8^+^ T cells, leading to partial suppression of tumor progression (65). In Nf1^−^/^−^ PTEN^−^/^−^ EGFRvIII^+^ mouse models, a single dose of anti-CD25NIB induces an IFN-γ-dependent remodeling of the tumor microenvironment, upregulates Fcγ receptor expression on myeloid cells, and enhances their phagocytic capacity. When combined with anti-PD-1 and an anti-EGFRvIII targeted antibody, complete tumor control can be achieved. This treatment also significantly depletes Tregs and activates CD8^+^ T cells in patient-derived glioblastoma tissue fragments, demonstrating strong potential for clinical translation (26).

Beyond direct targeting of Treg surface markers, immune checkpoint modulation offers a compelling alternative. An Fc-enhanced anti-CTLA-4 antibody, through its high affinity for FcγRIV, enables macrophages to selectively eliminate intratumoral Tregs while sparing peripheral Treg populations (66, 67). Preclinical studies indicate that combining FcE-aCTLA-4 with anti-PD-1, the chemotherapeutic agent doxorubicin, and ultrasound-mediated blood-brain barrier opening results in a cure rate of up to 90%, accompanied by infiltration of activated CD8^+^ T cells and the establishment of immune memory (65, 68). These findings underscore the robust immunomodulatory and antitumor efficacy of this combinatorial strategy (69).

Furthermore, targeting intracellular signaling pathways within Tregs offers a novel direction for immune modulation. The protease MALT1 plays a critical role in maintaining the immunosuppressive function of Tregs. Inhibiting its activity successfully reprograms intratumoral Tregs into effector cells with a pro-inflammatory phenotype, thereby augmenting local inflammatory responses (70). This reprogramming strategy exhibits significant synergy with anti-PD-1 therapy, jointly promoting antitumor immunity and improving tumor control. Notably, MALT1 inhibitors possess a dual mechanism of action, targeting both tumor cell proliferation and remodeling of the immunosuppressive microenvironment, and have now entered clinical trials (71).

### 3.2 Inhibition of Treg recruitment by blocking chemokine receptors

Inhibiting the trafficking of Tregs into the tumor microenvironment represents a promising immunotherapeutic avenue, with chemokine-receptor interactions serving as pivotal targets. Among these, CCR8 has garnered significant attention as a marker preferentially expressed by activated, intratumoral Tregs. The enrichment of CCR8^+^ Tregs in tumor tissues correlates with poor prognosis and enhanced immunosuppressive activity (28, 72). Therapeutically, Fc-optimized anti-CCR8 antibodies can selectively deplete this intratumoral Treg subset while sparing peripheral Tregs, thereby minimizing autoimmune risks and potentiating CD8^+^ T cell cytotoxicity (73, 74). Furthermore, the TNF-α/TNFR2/NF-κB axis, along with FOXP3, positively regulates CCR8 expression, suggesting that co-targeting TNFα signaling could augment the efficacy of anti-CCR8 therapies (28).

The CCR4 axis constitutes another key pathway for Treg recruitment. Tumor-derived ligands such as CCL22 and CCL2 engage CCR4 on Tregs, facilitating their migration and accumulation in malignancies including glioblastoma (32, 75). CCR4 blockades with antagonists or antibodies not only reduce Treg infiltration but also attenuates their suppressive function, thereby reinvigorating antitumor immunity-an effect observed in settings from solid tumors to chronic viral infections (33, 76). This strategy may exert broader immunomodulatory effects by disrupting a positive feedback loop between CCR4^+^ Tregs and TAMs, subsequently reducing TAM-driven pro-tumorigenic activities and metastasis, particularly after radiotherapy (33).

Emerging evidence also implicates CXCR3 in shaping the immunosuppressive niche. CXCR3^+^ Tregs are recruited to tumors via CXCL9 secreted by BATF3^+^ dendritic cells. Genetic ablation of CXCR3 disrupts this dendritic cell–Treg crosstalk, enhances antigen presentation to CD8^+^ T cells, and restrains tumor growth, highlighting CXCR3 blockade as a viable strategy to limit Treg-mediated suppression (77, 78).

Targeted inhibition of chemokine receptors such as CCR8, CCR4, and CXCR3 offers a rational and potent means to disrupt the spatial organization of immunosuppression within tumors. These approaches provide a compelling foundation for combination regimens aimed at overcoming immune resistance in refractory cancers like glioblastoma.

### 3.3 Functional modulation of Tregs: immune checkpoint inhibition and epigenetic regulation

The efficacy of ICIs, such as anti-PD-1 and anti-CTLA-4 antibodies, is profoundly shaped by the activity of Tregs within the tumor microenvironment. A nuanced understanding of how ICIs affect Tregs, alongside emerging insights into epigenetic and metabolic regulation, is critical for advancing combination immunotherapies.

ICI treatment exerts complex and often contrasting effects on Treg biology. Although anti-PD-1 therapy can reinvigorate CD8^+^ T cells, it may also paradoxically expand certain intratumoral Treg populations, thereby limiting therapeutic benefit (79). In contrast, anti-CTLA-4 antibodies can transcriptionally reprogram effector Tregs toward a less suppressive, naïve-like state, while simultaneously reversing CD8^+^ T cell exhaustion (80). The role of Tregs in ICI response is further complicated by contextual factors; in some non-small cell lung cancer patients, for instance, high FoxP3^+^ Treg density and TGF-β secretion have been linked to improved anti-PD-1 outcomes, suggesting a potential predictive role (81). ICIs can also attenuate Treg function by downregulating key molecules such as Foxp3, CD25, and IL-10 (82), and indirectly through gut microbiota-mediated metabolic shifts (83). However, these functional perturbations may contribute to immune-related adverse events, and adaptive resistance mechanisms frequently emerge, underscoring the complexity of the underlying Treg regulatory networks (84, 85).

Epigenetic mechanisms provide additional leverage for controlling Treg stability and function. Histone deacetylase (HDAC) inhibitors can disrupt Treg-suppressive circuits. The HDAC inhibitor SAHA interferes with the c-Myc/CCL1 axis, thereby alleviating immunosuppression and enhancing effector T cell activity. Similarly, the curcumin analog GO-Y030 suppresses the mTOR-S6 pathway in Tregs, reducing IL-10 production and improving anti-PD-1 efficacy (79). More broadly, epigenetic regulators directly influence the stability of FoxP3 and NF-κB, as well as their associated chromatin landscapes (86–88). Pharmacological modulation of these pathways via HDAC or other epigenetic inhibitors is therefore a promising strategy to functionally reprogram Tregs and improve immunotherapeutic outcomes (89, 90).

Combining metabolic intervention with ICIs represents another frontier for overcoming Treg-mediated resistance. Tumor-derived metabolites such as lactate can reinforce Treg-suppressive capacity; thus, disrupting their metabolic support systems may compromise Treg function (91). Genetic ablation of transketolase (TKT), a key enzyme in the pentose phosphate pathway, impairs Treg metabolism and function, even inducing autoimmunity (92). Pharmacological inhibition of phospholipid remodeling or mTOR signaling can similarly destabilize Treg homeostasis (93). When PI3K inhibitors are combined with PD-1 blockades, intratumoral Tregs are selectively reduced while CD8^+^ T cells are driven toward a memory phenotype, resulting in enhanced antitumor immunity (94). These findings suggest that simultaneous targeting of multiple immune checkpoints together with key metabolic regulators may help overcome resistance to monotherapy and achieve more durable clinical responses (95).

### 3.4 Combination strategies and novel platforms

Although strategies targeting regulatory Tregs show promise in glioblastoma treatment, the highly redundant and adaptable nature of the tumor’s immunosuppressive microenvironment often leads to resistance against single-agent therapies. Consequently, combining Treg-directed approaches with other treatment modalities or employing novel technological platforms has become a pivotal direction for enhancing efficacy and overcoming resistance (96).

Regarding combination with conventional therapies, radiotherapy, chemotherapy, and tumor-treating fields (TTFields) each demonstrate potential for synergy with immunomodulation (97). While radiotherapy can induce immunogenic cell death, it may also upregulate chemokines such as CCL22, paradoxically enhancing Treg recruitment to the tumor (21, 33). Therefore, combining radiotherapy with inhibitors of Treg recruitment or function modulators holds promise for augmenting anti-tumor immunity while counteracting radiotherapy-induced immunosuppression. Preclinical studies suggest this approach may improve local control and potentially elicit abscopal effects. Secondly, chemotherapeutic agents like temozolomide (TMZ) can influence lymphocyte populations beyond their direct cytotoxic effects. However, the broad lymphopenia they induce is a double-edged sword. A more refined strategy involves employing low-dose metronomic chemotherapy or selectively combining it with agents targeting Treg metabolism (98). This aims to undermine the survival advantage of Tregs within the tumor microenvironment while relatively enhancing effector T cell function (94). Finally, TTFields act by disrupting cell division, and emerging evidence suggests they may also modulate immune cell function. Although their specific impact on Tregs remains to be clarified, clinical exploration of TTFields combined with immunotherapy is underway. Future research needs to determine whether TTFields can regulate Treg migration or function, thereby providing a rationale for combination with Treg-targeting strategies.

In terms of combination with other immunotherapies and novel platforms, cutting-edge technologies like bispecific antibodies (bsAbs) and CAR-Tregs offer more precise control mechanisms. bsAbs can simultaneously bind two different antigens. For instance, antibodies could be designed to target both a Treg inhibitory receptor and a tumor-associated antigen, thereby confining Treg depletion or functional redirection to the tumor site and potentially reducing systemic autoimmunity risk (26). Furthermore, T cell engagers targeting CD3 and tumor antigens, primarily aimed at activating effector T cells, warrant exploration for sequential or combined use with Treg-targeting strategies. CAR-Treg therapy represents another emerging direction. By designing chimeric antigen receptors targeting GBM-associated antigens, Tregs can be guided to specifically infiltrate tumors. Such engineered cells could potentially be used to balance excessive inflammatory responses or combined with CAR-T cell therapy to manage cytokine storms. However, ensuring the functional stability of CAR-Tregs in a pro-tumor microenvironment and preventing their conversion to an effector phenotype pose significant challenges (16). Optimizing CAR signaling domains, combining them with epigenetic modulators, or employing novel receptor platforms like TRuC are potential strategies to enhance their stability (96).

Moreover, programmable cellular circuits like synthetic Notch (SynNotch) receptors allow for the design of logic-gated Tregs. For example, they can be engineered to activate only in the simultaneous presence of a tumor-specific antigen and a microenvironmental signal, thereby greatly enhancing treatment specificity and safety (15). *In vivo* reprogramming technology utilizes lipid nanoparticles (LNPs) to deliver mRNA encoding CARs or FoxP3 to specific T cell populations *in vivo*, enabling the direct generation of CAR-T or Treg-like cells. This bypasses complex ex vivo manufacturing processes and offers a more flexible therapeutic strategy for diseases like GBM that require long-term intervention (17).

**Figure 2:**
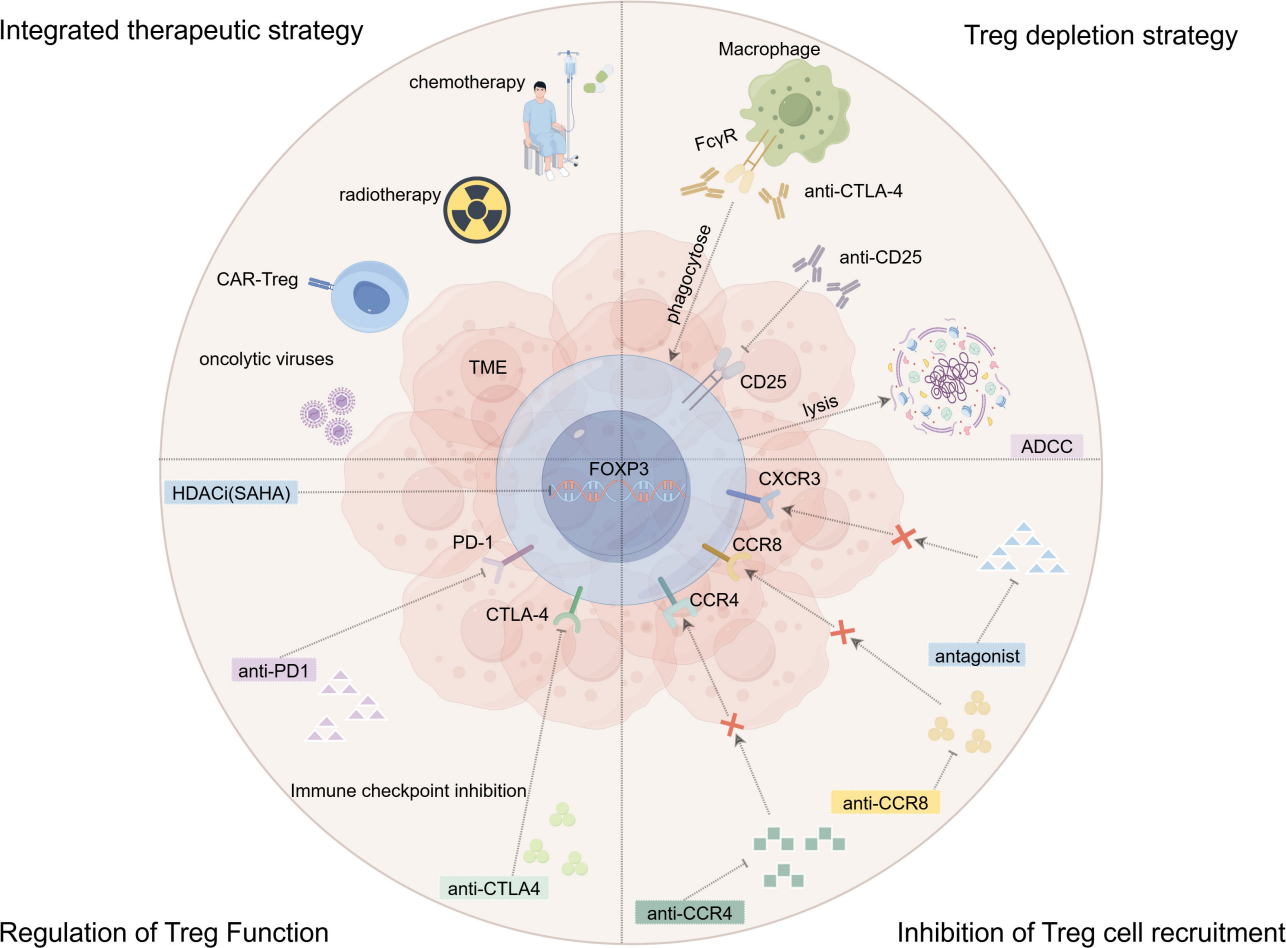
Overview of immunotherapeutic strategies targeting regulatory T cells in glioblastoma. Schematic representation of the major therapeutic approaches to counteract Treg-mediated immunosuppression in the tumor microenvironment (TME). These strategies can be broadly categorized into four groups: (1) Depletion of Tregs: Utilizing antibodies to target surface markers like CD25 or CTLA-4 for selective elimination. (2) Inhibition of Treg Recruitment: Blocking chemokine receptors such as CCR4, CCR8, and CXCR3 to prevent Tregs from trafficking into the TME. (3) Functional Modulation of Tregs: Employing immune checkpoint inhibitors (ICIs), epigenetic modulators (HDAC inhibitors), or metabolic interventions to disrupt Treg suppressive function and stability without immediate depletion. (4) Combination Therapies: Integrating Treg-targeting strategies with standard treatments (radiotherapy, chemotherapy) or other immunotherapies (anti-PD-1/PD-L1, cancer vaccines, oncolytic virotherapy, CAR-Treg) to achieve synergistic antitumor efficacy and overcome resistance. BBB, blood-brain barrier.

Looking ahead, the core of combination strategies lies in achieving spatiotemporal synergy and complementarity. Developing multimodal, individualized treatment regimens must be based on a deep understanding of the dynamic evolution of the GBM immune microenvironment. With the aid of multi-omics technologies and artificial intelligence, the organic integration of Treg-targeting therapies with the conventional treatments and emerging technological platforms holds promise for dismantling GBM’s formidable immunosuppressive fortress from multiple dimensions, ultimately leading to significant survival benefits for patients (**Figure 2**).

## 4 Clinical translation: challenges and prospects

### 4.1 Clinical trial progress and treatment outcomes

Preclinical studies and clinical trials have both contributed valuable insights into the efficacy of immunotherapies for GBM, though translating promising preclinical findings into clinical benefit remains a significant challenge. Despite the success of ICIs, such as anti-CTLA-4 and anti-PD-1 antibodies, across multiple cancer types, their efficacy as monotherapies in GBM remains limited. Clinical studies have not demonstrated a consistent overall survival benefit from ICI monotherapy in GBM patients, which can be attributed to the tumor’s low mutational burden, immunologically “cold” microenvironment, and specialized immunosuppressive mechanisms (99). For instance, in recurrent GBM, anti-PD-1 therapy failed to outperform bevacizumab in terms of survival (100). By contrast, combination approaches in preclinical models have yielded more promising outcomes. The anti-CTLA-4 antibody FcE-aCTLA-4, when administered alongside anti-PD-1, doxorubicin, and blood–brain barrier opening technology, achieved a 90% cure rate in a preclinical glioma model, accompanied by robust CD8^+^ T cell infiltration and durable immune memory (69). Similarly, dual blockade of PD-1 and BTLA in murine GBM models led to synergistic tumor control, prolonged survival, and reduced regulatory T cell numbers (99). These findings underscore the inadequacy of single-agent ICIs in reversing GBM-driven immunosuppression and highlight the potential of rationally designed combination immunotherapies, though these strategies now require rigorous evaluation in clinical settings.

Integrating metabolic modulators with ICIs represents another promising therapeutic avenue supported by preclinical evidence. Compounds such as PT2385 remodel the metabolic landscape of the tumor microenvironment, thereby enhancing immune activity and ICI responsiveness in preclinical models (40, 101). The natural polyphenol derivative pterostilbene (PTE) has been shown to induce GBM cell pyroptosis, suppress proliferation, and reverse immunosuppression by boosting CD8^+^ T cell and M1-like macrophage infiltration while reducing regulatory T cell frequency in murine glioma models, leading to markedly improved anti-PD-1 efficacy (102). Such strategies leverage both direct tumoricidal effects and immunomodulation of the microenvironment, offering a multifaceted basis for future clinical translation.

Underlying these therapeutic challenges are diverse and interconnected mechanisms of immunotherapy resistance in GBM. These include tumor cell plasticity, T cell exhaustion, recruitment of regulatory T cells and myeloid-derived suppressor cells (MDSCs), and crosstalk among multiple signaling pathways in the tumor niche (8, 103). For example, high FAT1 expression in the microenvironment not only drives tumor progression but also facilitates immune escape by enhancing immunosuppressive cell infiltration (104). In response, research is increasingly oriented towards personalized therapeutic strategies that account for individual tumor molecular profiles, immune microenvironment features, and resistance mechanisms to design multi-target combination regimens (105). Machine learning-based predictive models are also being developed to help identify patients most likely to benefit from specific immunotherapies, supporting a new paradigm of precision immuno-oncology for GBM (105).

### 4.2 Major hurdles: target specificity, treatment resistance, and toxicity clinical trial progress and efficacy evaluation

Although Tregs represent a promising therapeutic target in GBM due to their central role in sustaining an immunosuppressive microenvironment, several key challenges impede the clinical translation of Treg-directed strategies. These include limited targeting specificity, compensatory immune escape mechanisms, and difficulties in managing treatment-related toxicities.

A primary obstacle is the considerable phenotypic overlap between Tregs and conventional T cells, which complicates selective targeting. While Tregs in GBM consistently express canonical markers such as CD25 and FoxP3, these molecules are also present on recently activated effector T cells. Consequently, anti-CD25 antibodies may deplete Tregs and expand CD8^+^ T cells but can simultaneously impair effector T cell responses, leading to unintended immunosuppression or toxicity (26). Similarly, receptors like GITR, though highly expressed on Tregs and amenable to agonistic targeting, play complex roles in T cell homeostasis. Agonists aimed at attenuating Treg function must be carefully tuned to avoid disrupting broader immune regulation (21, 106). Thus, developing tools capable of discriminating Tregs from other T cell subsets-whether through novel surface markers, functional states, or activation context, is essential for achieving both efficacy and safety.

A second challenge arises from the resilience and adaptability of the GBM immunosuppressive network, which often leads to monotherapy resistance. Tregs employ multiple mechanisms to sustain local tolerance, including secretion of inhibitory cytokines and upregulation of immune checkpoints such as PD-1 and CTLA-4 (104, 107). These are further reinforced by stromal and myeloid components, which express ligands like PD-L1 and CD47 (108), collectively establishing a robust barrier to T cell-mediated attack (98, 109). Moreover, conventional treatments such as radiotherapy can paradoxically enhance Treg infiltration, thereby limiting the efficacy of accompanying immunotherapies (21, 110). Given the marked intertumoral heterogeneity in GBM and the dynamic nature of its immune landscape, successful intervention will likely require multimodal regimens that simultaneously disrupt complementary immunosuppressive pathways.

Finally, managing immune-related adverse events remains a critical hurdle in clinical development. Treg-directed therapies risk breaking self-tolerance, potentially triggering autoimmunity or uncontrolled inflammation (84, 85). The primary concern is the disruption of systemic immune homeostasis. Since Tregs are pivotal in maintaining peripheral tolerance, their non-specific or systemic depletion can precipitate immune-related adverse events akin to those observed with checkpoint inhibitors, such as colitis, dermatitis, and endocrinopathies (84). This risk is compounded within the unique context of the central nervous system (CNS), where disrupting the delicate immunoregulatory balance may theoretically exacerbate neuroinflammation or compromise blood-brain barrier integrity (111). Furthermore, the issue of target specificity is paramount; for instance, anti-CD25 antibodies may inadvertently affect activated effector T cells that transiently express CD25, potentially undermining antitumor immunity while exacerbating toxicity (26). Therefore, future strategies must prioritize enhancing specificity for intratumoral Treg subsets, exploring localized delivery methods, and optimizing dosing schedules to maximize the therapeutic window by balancing potent antitumor immunity with the preservation of systemic immune homeostasis. This is particularly evident with broad-targeting approaches such as anti-CD25 antibodies, which may cause systemic immune dysregulation (26). Combination strategies, for example, dual checkpoint blockade-often enhance anti-tumor efficacy but also increase the frequency and severity of immune-mediated toxicities (112). In GBM, the unique anatomy of the central nervous system and the presence of the blood-brain barrier further complicate drug delivery and toxicity management, especially when combining immunotherapy with radiotherapy (111). Moving forward, improving safety will require advances in toxicity monitoring, early intervention protocols, and targeted delivery systems, such as nanocarriers or viral vectors-that maximize intratumoral activity while sparing systemic immunity (113). **Supplementary Table 1** provides an overview of current clinical trials targeting Tregs and offers directions for developing Treg-targeting strategies in GBM.

### 4.3 Evolution of Treg-targeting strategies: a historical and strategic perspective

During this period (Early Stage,2010-2015), research on Treg-targeting strategies primarily focused on proof-of-concept validation. The main approach involved using non-specific antibodies to deplete Treg cells, aiming to enhance anti-tumor immune responses (114). However, these strategies faced significant challenges: systemic Treg depletion led to severe autoimmune toxicity, and targeting specificity was poor, particularly in “cold tumors” such as GBM, where efficacy was limited. Representative studies gradually recognized that systemic Treg depletion was not feasible, necessitating further exploration of tumor-specific targets to avoid systemic immune imbalance (115).

With deepening research (Intermediate Stage,2016-2020), the strategic focus shifted toward precision and combination therapies. Scholars began exploring targets enriched in tumors (such as CCR4 and CCR8) to enhance the specificity of Treg depletion (116). Additionally, the combination of Treg-targeted therapies with immune checkpoint inhibitors or standard treatments became a mainstream direction (117). Nevertheless, this stage still faced key challenges: the emergence of drug resistance, the complexity of the immune microenvironment, and the additive toxicity associated with combination therapies. Representative studies further revealed the high heterogeneity and plasticity of Tregs in GBM and began investigating novel regulatory mechanisms, such as metabolic and epigenetic pathways, laying the foundation for subsequent strategy optimization.

The current research (Current Stage,2021-Present) focus has shifted toward next-generation technologies and personalized treatments. Engineered cell therapies (CAR-Treg), bispecific antibodies, and *in vivo* reprogramming technologies have entered the exploratory stage, with an emphasis on biomarker-based patient stratification to improve treatment precision. However, this stage still faces core challenges: issues related to the persistence and safety of engineered cells, overcoming the extreme immunosuppression of TME, and identifying biomarkers predictive of therapeutic efficacy. Representative studies include the initiation of the first CAR-Treg clinical trials and the development of highly specific antibodies targeting molecules such as CCR8 (114).

### 4.4 Strategies to overcome the blood–brain barrier in GBM immunotherapy

The blood–brain barrier (BBB) represents a unique anatomical and physiological obstacle for GBM immunotherapy, severely limiting the delivery of systemically administered antibodies, small molecules, and immune cells to the tumor microenvironment (118). Composed of tight junctions, efflux transporters, and enzymatic barriers, the BBB restricts over 98% of small-molecule drugs and nearly all large-molecule biologics from reaching therapeutic concentrations within brain tumors (119). Recognizing this fundamental challenge, several complementary strategies have been developed to enhance BBB penetration specifically for GBM treatment.

Focused ultrasound (FUS) combined with intravenous microbubbles enables transient, targeted BBB disruption. FUS exposure causes microbubble oscillation, mechanically opening tight junctions and allowing passive passage of circulating therapeutic agents (120). In preclinical GBM models, FUS-mediated BBB opening has been shown to enhance intratumoral accumulation of anti-PD-1 antibodies by 5- to 10-fold, improve CD8^+^ T cell infiltration, and augment antitumor immune responses (121). Early-phase clinical trials have demonstrated the safety and feasibility of FUS-mediated BBB opening in GBM patients, with successful drug delivery confirmed by imaging and improved progression-free survival observed in some cohorts (68).

Intracranial locoregional delivery strategies circumvent the BBB entirely by administering therapeutics directly into the tumor resection cavity or brain parenchyma. Convection-enhanced delivery (CED) utilizes positive pressure to infuse agents over large volumes, achieving high local concentrations while minimizing systemic exposure (122). Preclinical studies have demonstrated that CED of immune checkpoint inhibitors, including anti-CTLA-4 and anti-PD-1 antibodies, induces robust antitumor immunity and long-term survival in murine GBM models (123). Clinical evaluation of CED for GBM has primarily focused on chemotherapeutics and targeted toxins, with ongoing trials exploring immunotherapeutic agents.

Nanocarrier-based systems offer a versatile platform for enhancing BBB penetration through active targeting mechanisms. Lipid-based nanoparticles, polymeric nanoparticles, and exosomes can be engineered with surface modifications, such as conjugation to transferrin, low-density lipoprotein receptor, or glucose transporters—to facilitate receptor-mediated transcytosis across the BBB (124). In preclinical GBM models, nanoparticle-encapsulated immune modulators, including toll-like receptor agonists and checkpoint inhibitors, have demonstrated 3- to 5-fold improved brain penetration, reduced systemic toxicity, and enhanced antitumor efficacy compared to free drugs (125) Additionally, some nanocarriers exploit the enhanced permeability and retention (EPR) effect in regions where the BBB is compromised by tumor growth, providing passive tumor targeting (126). Each strategy presents distinct advantages and limitations; combining these approaches may offer the greatest potential for overcoming the BBB and realizing the full potential of immunotherapy for GBM.

## 5 Future perspectives and directions

Targeting Tregs represents a promising avenue to reverse immunosuppression in GBM. Future advances will likely stem from integrated strategies that combine mechanistic insights with innovative technologies to achieve precise and effective Treg modulation.

Mechanism-driven approaches will form the foundation of next-generation therapies. By interfering with Treg metabolic reprogramming, epigenetic modifications, and key intracellular signaling pathways, it may be possible to systematically disrupt their immunosuppressive functions without broad immune cell depletion. These efforts will be complemented by progress in antibody engineering, such as bispecific antibodies and near infrared photoimmunotherapy, which can improve the specificity of intratumoral Treg targeting. Coupled with small-molecule inhibitors, such technologies are poised to enhance local Treg control while preserving systemic immune homeostasis.

Novel delivery systems will also be essential to overcome anatomical and physiological barriers in GBM. Nanocarriers, including melanin-based nanoparticles and biomimetic vesicles, as well as localized sustained-release platforms may help concentrate immunomodulatory agents within the tumor area. These systems are expected to enhance antitumor immunity while reducing systemic exposure, thereby widening the therapeutic window and mitigating off-target toxicity.

Finally, precision medicine will increasingly guide Treg-targeted immunotherapy. The integration of multi-omics profiling, such as single-cell sequencing, spatial transcriptomics, and radiogenomics, will help characterize patient-specific Treg subsets and immune microenvironmental features. Artificial intelligence (AI) tools will further assist in stratifying patients, selecting optimal combination therapies, and predicting treatment-related risks based on clinical and molecular datasets.

The future of Treg-targeted therapy in GBM lies in a holistic framework that unites mechanistic discovery, technological innovation, and individualized clinical application. Such an integrated approach offers a promising path to overcome current limitations in GBM immunotherapy and move closer to truly precision immuno-oncology.

## 6 Conclusion

Regulatory T cells are established as pivotal mediators of immunosuppression in the glioblastoma microenvironment, rendering their therapeutic targeting a rational and compelling immunotherapeutic strategy. Current modalities, such as Treg-depleting antibodies, checkpoint inhibition, and metabolic interference, have shown encouraging potential in reversing local immune suppression. However, achieving tumor-selective targeting while preserving systemic immune homeostasis remains a central challenge.

The integration of Treg-focused interventions with standard therapies and complementary immunomodulatory agents holds promise for overcoming resistance and enhancing treatment efficacy. Future success will depend on a more nuanced understanding of Treg plasticity and subset-specific functions within the GBM context, supported by advanced biomarker development and patient stratification. Through continued multidisciplinary collaboration and mechanistic innovation, Treg-targeted approaches are expected to evolve into clinically meaningful components of combination regimens, ultimately contributing to more effective and durable control of this devastating disease.
